# Hydrolysed protein enteral nutrition is not superior to polymeric whole protein feeding with regard to gastrointestinal feeding tolerance and feeding adequacy

**DOI:** 10.1186/s13054-017-1817-8

**Published:** 2017-09-05

**Authors:** Arthur R. H. van Zanten, Gunnar Elke

**Affiliations:** 1 Department of Intensive Care, Gelderse Vallei Hospital, Willy Brandtlaan 10, 6716 RP Ede, The Netherlands; 2 Department of Anaesthesiology and Intensive Care Medicine, University Medical Centre Schleswig-Holstein, Campus Kiel, Arnold-Heller-Str. 3 Haus 12, D-24105 Kiel, Germany

We would like to comment on the SPIRIT trial by Jakob and co-workers comparing the effects of hydrolysed protein enteral nutrition (EN; Peptamen AF®) and isocaloric control polymeric whole protein feed (Isosource® Energy) on gastrointestinal feeding tolerance, including diarrhoea and feeding adequacy [1]. No differences in diarrhoea-free days and number of diarrhoea events were observed.

In a recent meta-analysis [2] only 121 patients from four studies were included, 63 in peptide-based groups and 58 patients in control arms. Combining these data with the SPIRIT trial results (N = 211 patients), no benefits with respect to diarrhea incidence during intensive care unit (ICU) stay and feeding adequacy are observed in favour of peptide-based EN (Table 1).

**Table 1:** Randomized controlled trials addressing diarrhoea frequency in critically ill patients associated with enteral feeds

Diarrhoea		Peptide-based EN			Polymeric whole protein EN		
Study	Year	Events (n)	Percentage	Total (n)	Events (n)	Percentage	Total (n)
Brinson (as in [2])	1988	1	14.3	7	3	60.0	5
Meredith (as in [2])	1990	0	0.0	9	4	44.4	9
Mowatt-Larssen (as in [2])	1992	6	28.6	21	6	30.0	20
Heimburger (as in [2])	1997	10	38.5	26	4	16.7	24
Jakob et al. [1]	2017	29	63.0	46	31	70.5	44
Total		46	42.2	109	48	47.1	102

We disagree with the last part of the authors’ conclusion: “While the data of this pilot study do not indicate that modification of the protein and fat content can attenuate the incidence of diarrhea, it does show that a product like Peptamen® AF can effectively deliver a high daily protein amount without overfeeding the ICU patients.” Both feeds are isocaloric but Peptamen® AF delivers 25%, and Isosource® Energy 16% of energy by proteins. Per calorie administered, the protein dose is higher (25/16 × 100% = 56.3%) in the Peptamen® AF group.

When gastrointestinal tolerance is similar, using the same caloric targets means protein intake in the Peptamen® AF should at least be 56% higher.

Protein intake was higher but the difference was lower than expected (1.13/0.80 = 41%). Furthermore, the accumulated caloric deficit difference was significantly larger in the Peptamen® AF group (*P* < 0.014). Thus, higher protein intake in the Peptamen® AF group is mainly due to differences in product composition and not due to better gastrointestinal tolerance.

The authors relate differences in caloric intake to more stoppages in the Peptamen® AF group; however, this post-hoc observation must be qualified considering that interruptions of EN are also related to gastrointestinal tolerance and the inability to deliver EN to achieve prescribed targets is part of the definition of feeding intolerance [3]. The claim by the authors can only be substantiated when an isocaloric and isonitrogenous control feed is used and protein delivery is better in the peptide-based feeding arm. The SPIRIT trial does not answer this.

In the absence of any benefits on EN tolerance or diarrhoea, and considering higher costs of hydrolysed protein feeds, we feel supported by recent guidelines recommending use of a polymeric formula when initiating EN in critically ill patients [4].

## Abbreviations

EN: Enteral nutrition
ICU: Intensive care unit

## References

[1] Jakob SM, Bütikofer L, Berger D, Coslovsky M, Takala J (2017). A randomized controlled pilot study to evaluate the effect of an enteral formulation designed to improve gastrointestinal tolerance in the critically ill patient-the SPIRIT trial. Crit Care.

[2] Canadian Clinical Practice Guidelines: 4.3 Strategies for optimizing and minimizing risks of EN: whole protein vs. peptides. http://criticalcarenutrition.com/docs/CPGs%202015/4.3%202015.pdf. Accessed June 14, 2017.

[3] Reintam Blaser A, Malbrain ML, Starkopf J (2012). Gastrointestinal function in intensive care patients: terminology, definitions and management. Recommendations of the ESICM Working Group on Abdominal Problems. Intensive Care Med.

[4] McClave SA, Taylor BE, Martindale RG (2016). Guidelines for the provision and assessment of nutrition support therapy in the adult critically ill patient: Society of Critical Care Medicine (SCCM) and American Society for Parenteral and Enteral Nutrition (A.S.P.E.N.). J Parenter Enteral Nutr.

